# Biphasic Epithelial Predominant Synovial Sarcoma Presenting as Painful Thigh Mass

**DOI:** 10.30699/ijp.2019.90673.1865

**Published:** 2019-08-01

**Authors:** Elham Mirzaian, Seyed Mohammad Tavangar, Sahar Montazeri, Farbod Emami Yeganeh

**Affiliations:** 1 Department of Pathology, Shariati Hospital, Tehran University of Medical Sciences, Tehran, Iran; 2 Department of Pathology, Tehran University of Medical Sciences, Tehran, Iran; 3 Shaheed Beheshti University of Medical Sciences**, **Tehran, Iran

**Keywords:** Synovial sarcoma, Epithelial predominant, Thigh mass

## Abstract

Synovial sarcomas are soft tissue neoplasms mostly located in the lower extremities of young adults.

A case of synovial sarcoma of the thigh in a 35-year-old male with the predominant epithelial component is reported. Microscopically the tumor showed variable-sized well-differentiated glands lined by the cuboidal cells with small foci of spindle cell component between glandular structures. Immunohistochemically glandular components showed positivity for the pan CK and EMA while CD99 and TLE1 were positive in both glandular and spindle cell components.

This type of synovial sarcoma could be indistinguishable from metastatic adenocarcinoma and malignant adnexal tumor, thus, immunohistochemistry and molecular studies play an essential role in the exact diagnosis of this type of tumor.

## Introduction

Synovial sarcoma is a distinct aggressive neoplasm that occurs most often in teenagers and young adults (mean age:35) (1). The etiology of this tumor is unknown, but some authors believe that it arises from multi-potential stem cells with differentiation into the epithelial or mesenchymal structures (2, 3). The most common clinical findings are palpable and deep-seated mass associated with the pain or tenderness (4). There are no known risk factors for this tumor (7), but some patients had a history of trauma (4-7). Mostly lower extremities are affected by the tumor (8), and involvement of the articular space is rare (4). Most cases occur in juxta-articular areas (8). This tumor is classified histologically into biphasic, monophasic and poorly differentiated types (4, 5). The pure epithelial pattern is rare (2, 4) and the two major differential diagnosis for the epithelial predominant synovial sarcoma is metastatic adenocarcinoma and malignant adnexal tumor (2, 4). Therefore, an exact diagnosis of this subtype of synovial sarcoma, identifying minute foci of spindle cells and cytogenetic or molecular findings [t (X,18)], are necessary (4).

## Case Report

**Figure 1:**
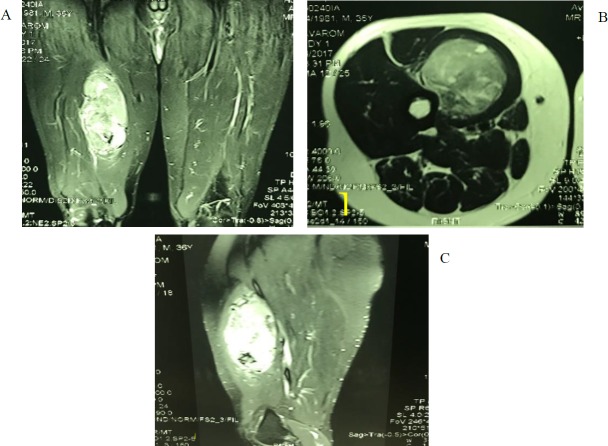
A) AP, B) transverse and C) lateral view of right femur MRI showing large lobulated hyper-vascular mass lesion with the diameter of 16.5X7cm in deep portion of the right rectus femoris muscle

**Figure 2:**
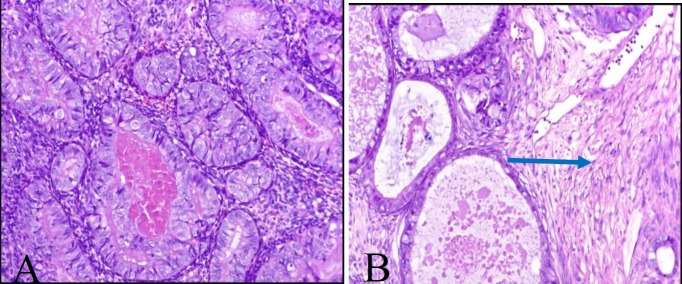
A) shows epithelial component of the tumor composed of glandular structures lined by the cuboidal to columnar cells ( H&E, 200X) and B) shows foci of spindle cell component composed of monotonous spindle cells arranged in fascicle – Pointed by blue arrow ( H&E, 200X)

**Figure 3:**
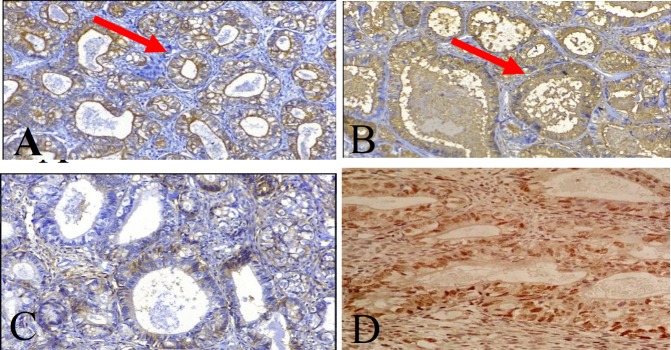
IHC showing A, B) PanCK and EMA positivity in epithelial component -Pointed by red arrows (IHC 200X), C) CD99 positivity in epithelial and spindle component (IHC 200X), D) Nuclear staining for TLE1 in both epithelial and spindle cell components (IHC 200X)

A 35-year-old male referred to our center with a painful thigh mass. He had first noticed the mass 8 years ago after minor trauma. The tumor was growing slowly during these years, until about 6 months ago that started to grow rapidly and became painful. On physical examination, a 10 cm, well-defined, firm movable and mildly tender mass was palpated in the medial aspect of the right thigh. Magnetic resonance imaging (MRI) revealed a large lobulated hypervascular mass lesion in the deep portion of the right rectus femoris muscle (Fig. 1). Computed tomography scan of the abdomen, pelvis and whole body bone revealed no pathologic findings. The patient was planned for the incisional biopsy. A wedge-shaped portion of the tumor was removed and sent for the pathologic examination. Macroscopically, the specimen consisted of multiple fragments of creamy-tan soft tissue totally measuring 5x3x1 cm and was embedded entirely. Microscopically, the tumor showed variable-sized well-differentiated gland-like structures lined by the cuboidal cells with clear to pinkish cytoplasm. Some of these glands contained intraluminal eosinophilic material (Fig. 2A). Small foci of spindle cells arranged in the fascicles were also identified between glandular structures (Fig. 2B). Immunohistochemically, the glandular components showed strong reactivity for pan CK, CK7, CK19 and EMA (Fig. 3A&B). CD99 was positive in both spindle and epithelial components (Fig. 3C). Bcl2 was only positive in spindle cells, and CD34 was negative in both components. The TLE1 was positive in both epithelial and spindle cell components (Fig. 3D). The diagnosis was done for the epithelial predominant synovial sarcoma.

## Discussion

Synovial sarcoma is an aggressive neoplasm that occurs at any age but it is most prevalent in teenagers and young adults.

In spite of its name, the involvement of joint cavities and the synovial membrane is rare (5, 9) and this tumor occurs in para-articular areas. The more common sites of involvement are knee and ankle (2).This tumor is associated with the t(X,18) (p11;q11) chromosomal translocation (7, 10, 11). Histologically synovial sarcoma can be classified into: 1) biphasic type, including distinct epithelial and spindle cell component, 2) monophasic, including the fibrous type and epithelial type and, 3) poorly differentiated type (4, 5). Biphasic (epithelial predominant) synovial sarcoma is rare and the most important differential diagnosis for this type is metastatic adenocarcinoma and malignant adnexal tumor (2, 9). In this type, small foci of spindle cell differentiation and evidence of the cytogenetic or molecular genetic data that are characteristic for the synovial sarcoma is helpful for the diagnosis of this rare entity (4).

Immunohistochemical analysis and finding the specific chromosomal translocation is necessary for the exact diagnosis of this tumor. Most synovial sarcoma show immunoreactivity for the cytokeratins (specially CK7 and CK19) and EMA. There is focal positivity for S100 and immunoreactivity for CD99 and CD56 (5). TLE1 is a useful marker for the diagnosis of synovial sarcoma, especially in CK negative cases. An important negative IHC marker in synovial sarcoma is CD34 (4).

In a case report by Ishida T et al., a case of synovial sarcoma with predominant epithelial component was reported. In this case, they stated that presence of a biphasic pattern, even with few scattered spindle cell component has an essential role in the correct diagnosis of synovial sarcoma and its distinguish from carcinoma (12).

In another case report by Salgaonkar G et al., a case of synovial sarcoma with the primary manifestation of the chronic non-healing ulcer of the foot was presented. By histopathologic examination and IHC study of this case, the diagnosis of epithelial predominant biphasic synovial sarcoma was made (1).

Synovial sarcoma is aggressive soft tissue tumor with poor prognosis (2, 5) . Some indicators of the adverse prognosis are tumor size more than 5 cm, truncal location, male gender, high nuclear grade and presence of poorly differentiated areas (2, 4). The optimal and choice treatment for the synovial sarcoma is complete surgical excision with removal of the adequate margins (2, 4, 5). Local radiotherapy and chemotherapy can be helpful, especially in the high-risk patients (2).

## Conclusion

We report a rare case of predominant epithelial synovial sarcoma in a young man presenting as painful thigh mass with the previous history of minor trauma. It is considered that this tumor could be mistaken with other malignant tumors such as metastatic adenocarcinoma, malignant adnexal tumor, and malignant melanoma. Thus, the role of immuno-histochemistry and molecular study for the exact diagnosis of this tumor is important.
